# Elevated plasma pyruvate kinase M2 concentrations are associated with the clinical severity and prognosis of coronary artery disease

**DOI:** 10.11613/BM.2024.010704

**Published:** 2023-12-15

**Authors:** Zi-wen Zhao, Yi-wei Xu, Xin-tao Zhang, Hang-hao Ma, Jing-kun Zhang, Xue Wu, Yu Huang

**Affiliations:** 1 Department of Cardiology, Fujian Heart Medical Center, Fujian Institute of Coronary Heart Disease, Fujian Medical University Union Hospital, Fujian Medical University, Fuzhou, China; 2 Department of Cardiology, Ningde People Hospital, Ningde, China; 3 Cardiovascular Research Institute, University of California, San Francisco, USA; 4 Institute for Global Health Sciences, University of California, San Francisco, USA

**Keywords:** biochemistry, cardiovascular diseases, coronary stenosis, pyruvate kinase, inflammation

## Abstract

**Introduction:**

Pyruvate kinase M2 (PKM2) was involved in the pathophysiology of atherosclerosis and coronary artery disease (CAD). We tested whether plasma PKM2 concentrations were correlated with clinical severity and major adverse cardiovascular events (MACEs) in CAD patients.

**Materials and methods:**

A total of 2443 CAD patients and 238 controls were enrolled. The follow-up time was two years. Plasma PKM2 concentrations were detected by enzyme-linked immunosorbent assay (ELISA) kits (Cloud-Clone, Wuhan, China) using SpectraMax i3x Multi-Mode Microplate Reader (Molecular Devices, San Jose, USA). The predictors of acute coronary syndrome (ACS) were assessed by logistic regression analysis. The association between PKM2 concentration in different quartiles and MACEs was evaluated by Kaplan-Meier (KM) curves with log-rank test and Cox proportional hazard models. The predictive value of PKM2 and a cluster of conventional risk factors was determined by Receiver operating characteristic (ROC) curves. The net reclassification improvement (NRI) and the integrated discrimination improvement (IDI) were utilized to evaluate the enhancement in risk prediction when PKM2 was added to a predictive model containing a cluster of conventional risk factors.

**Results:**

In CAD patients, PKM2 concentration was the independent predictor of ACS (P < 0.001). Kaplan-Meier cumulative survival curves and Cox proportional hazards analyses revealed that patients with a higher PKM2 concentration had higher incidence of MACEs compared to those with a lower PKM2 concentration (P < 0.001). The addition of PKM2 to a cluster of conventional risk factors significantly increased its prognostic value of MACEs.

**Conclusion:**

Baseline plasma PKM2 concentrations predict the clinical severity and prognosis of CAD.

## Graphical abstract

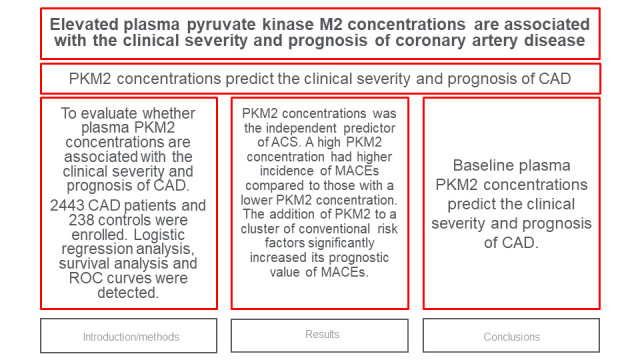

## Highlights

Pyruvate kinase M2 is a predictor of clinical severity of coronary artery disease

Pyruvate kinase M2 predicts the incidence of major adverse cardiovascular events

Pyruvate kinase M2 showed additional prognostic value in coronary artery disease

## Introduction

Coronary artery disease (CAD) is one of the main causes of death worldwide, necessitating effective risk stratification strategies for early identification and intervention (*1*). Traditional risk factors, such as age, gender, hypertension, and hyperlipidemia, have been widely used in clinical practice for CAD risk assessment. However, these factors may not fully capture the complexity and variability of individual risk profiles. Therefore, there is a need to explore additional biomarkers that can improve risk prediction and refine the current risk stratification models. Several biomarkers such as troponin I and T are well established and commonly used in clinical practice (*2*). However, few biomarkers are accessible for predicting the severity of coronary stenosis and clinical outcomes in CAD patients.

Pyruvate kinase M2 (PKM2) is a key metabolic enzyme regulating the final rate-limiting step of glycolysis by the formation of pyruvate and adenosine triphosphate (ATP) from phosphoenolpyruvate (PEP) and adenosine diphosphate (ADP) (*3*). Additionally, PKM2 has been reported to function as a protein kinase to regulate many other cellular functions, such as gene transcription and cell cycle progression (*4*, *5*). It is over-expressed in a broad range of human cancers and associated with poor clinical outcomes (*6*, *7*). Pyruvate kinase M2 can be highly secreted to blood plasma of patients and elucidates its potential use as a circulating peripheral biomarker (*8*-*10*).

A recent study has shown that PKM2 regulates cholesterol uptake and foam cell formation in macrophages stimulated with lipopolysaccharides or oxidized low-density lipoprotein (ox-LDL) (*11*). Pyruvate kinase M2 is also upregulated in monocytes/macrophages and links metabolic and inflammatory dysfunction in patients with atherosclerotic CAD (*12*). Given the potential role of PKM2 in the pathogenesis of CAD, we hypothesized that plasma PKM2 concentrations would be associated with the clinical severity of CAD and the occurrence of major adverse cardiovascular events (MACEs) in CAD patients. Furthermore, we aimed to evaluate the utility of PKM2 as a complementary biomarker to conventional risk factors in the risk stratification of CAD.

## Materials and methods

### Study population

The prospective observational study is conducted in Fujian Medical University Union Hospital. From June 2018 to June 2020, 2443 consecutive CAD patients undergoing coronary angiography (CAG) during hospitalization and consented to be followed for 2 years were recruited. At the same period, 238 age- and sex-matched patients undergoing CAG the hospital due to precardiac discomfort and no apparent angiographical abnormality (no stenosis > 20%) were enrolled as controls (*13*). Coronary artery disease was defined as the presence of angiographically documented diameter stenosis of at least 50% in one of the major coronary arteries or their main branches (*14*). Patients with CAD were divided into two subgroups: patients with chronic coronary disease (CCS) and patients with acute coronary disease (ACS). Diagnosis was made according to the standard definitions of the European Society of Cardiology and the American College of Cardiology, respectively (*15*, *16*). Patients were excluded if they had acute ST-segment elevation myocardial infarction (MI) within 72 h, suspected myocarditis or pericarditis, active or chronic inflammatory and autoimmune disease, end-stage liver cirrhosis or severe kidney disease and undergoing dialysis, malignancy, symptomatic peripheral vascular disease or stroke.

### Study protocol

The baseline and clinical characteristics including demographic data, body mass index (BMI), laboratory indices, medication and risk factors were gathered at study entry. Patients were given standard medications (*i.e*. anti-platelet agents, statins, beta blockers *etc.*) according to the up-to-date guidelines during the 2-year follow-up period. The primary end-point of MACEs include death due to any cause, nonfatal acute myocardial infarction, target vessel revascularization (TVR) and readmission for revascularization therapy due to angina pectoris. Hospital readmissions and clinical outcomes were identified through electronic medical records or telephone interviews with the patients or relatives.

Written informed consent was obtained from all patients. The study protocol was conducted in accordance with the Declaration of Helsinki and approved by the ethics committee of Fujian Medical University Union Hospital (Approval NO.2018KY016).

### Definition of cardiovascular risk factors

Hypertension refer to blood pressure ≥ 140/90 mmHg or using antihypertensive drug therapy. Diabetes mellitus (DM) refer to fasting blood glucose (FBG) concentration ≥ 7.0 mmol/L or glycosylated hemoglobin (HbA1c %) value ≥ 6.5% or non-fasting blood glucose concentration ≥ 11.1 mmol/L or receiving hypoglycemic treatments. Smoking status refer to at least one cigarette/day and lasting more than six months (*14*).

### Laboratory measurements

Fasting blood samples were obtained from all patients prior to CAG. Blood samples were collected using specific tubes for different purposes. Ethylenediaminetetraacetic acid (EDTA)-containing plasma tubes (Becton Dickinson, Franklin Lakes, USA) were used for complete blood count (CBC) and PKM2 detection, while lithium heparin plasma tubes (Becton Dickinson, Franklin Lakes, USA) were used for N-terminal pro-brain natriuretic peptide (NT-proBNP) detection. Serum tubes without any anticoagulant (Becton Dickinson, Franklin Lakes, USA) were used for other routine biochemistry tests. The collected blood samples for PKM2 were centrifuged at 2560xg for 15 minutes, allowing for the extraction of plasma samples. These plasma samples were subsequently stored at a temperature of - 80 °C until they were ready for analysis. It should be noted that the storage duration of the serum samples did not exceed one month to ensure the preservation of sample quality. Routine biochemical indicators were detected by an automatic biochemical analyzer LX-20 (Beckman Coulter, Brea, USA). These indicators include total cholesterol (TC), high/low density lipoprotein cholesterol (HDL-c/LDL-c), triglyceride (TG), creatinine (Crea), blood urea nitrogen (BUN), uric acid (UA) and FBG. Complete blood count for white blood cell (WBC) count was measured by Sysmex XN-3000 hematology analyzer (Sysmex Corporation, Kobe, Japan). NT-proBNP was measured by Cobas 6000 analyzer (Roche, Basel, Switzerland). High-sensitivity CRP (hs-CRP) was measured by an Immage 800 Immunochemistry System (Beckman Coulter, Brea, USA). Homocysteine (Hcy) was measured by Abbott i2000SR (Abbott Laboratories, Abbott Park, USA). Plasma PKM2 was measured by enzyme-linked immunosorbent assay (ELISA) Kits (NO. L181016633, Cloud-Clone, Wuhan, China) according to the manufacturer’s instructions. The ELISA procedure involves adding diluted standard, blank, and samples to wells, incubating them, adding detection reagents, washing the wells, adding substrate solution for color change, and finally measuring the plate using SpectraMax i3x Multi-Mode Microplate Reader (Molecular Devices, San Jose, USA) at 450 nm. Quality control measurement of the ELISA kits including reagent quality control, performance validation, standard curve generation, and inter/intra-assay variability assessment were performed in our laboratory. The intra-and inter-assay coefficients of variation were 7.65% and 9.82%, respectively. The average recovery rate was 86%. The linearity of the kit ranges from 90-103%. The provided measurements suggested that the ELISA kits used in our laboratory have good precision, reproducibility, and accuracy within the specified ranges. The ELISA samples were tested in duplicate and the determinations were averaged.

### CAG and echocardiogram

All the patients underwent CAG performed following standard techniques. Angiographic views were obtained after administration of intracoronary nitrate (100 or 200 μg). The results of CAG were evaluated by two blinded interventional cardiologists. Angiographic severity of CAD was assessed by the SYNergy between percutaneous coronary intervention with TAXus and cardiac surgery (SYNTAX) score, which is well established and validated in many studies (*17*). The echocardiogram was performed by a Vivid 3 model echocardiography device (GE HealthCare, Chicago, USA).

### Statistical analysis

The study sample size was calculated using power analysis with the following assumptions: an expected MACEs rate of 15% in CAD patients, a 0.2 ng/mL difference in mean PKM2 concentration between patients with MACEs and without MACEs, using a significance level of 0.05 and power 0.9. Therefore, at least 82 MACEs were needed (*18*). The distribution of continuous variables was tested by the Kolmogorov-Smirnov test. Age was reported as median (min - max). Other continuous variables not following normal distribution were presented using median and interquartile range. Nominal variables were described using numbers and percentages. Mann-Whitney U test and Chi-square test was explored to examine between-group differences. Kruskal-Wallis test and Chi-square was used for comparisons among multiple groups. The predictors of ACS in CAD patients were assessed by univariate and multivariate logistic regression. The correlation between PKM2 concentrations and SYNTAX scores was assessed by Spearman correlation analysis. The independent predictors of log (SYNTAX scores) were assessed by multivariate linear regression analysis. The survival curves for PKM2 concentration in different quartiles was assessed by Kaplan-Meier (KM) analysis with log-rank test. The association between PKM2 concentration in different quartiles and MACEs was assessed by Cox proportional hazard models after adjustment for potential confounding covariates. Receiver operating characteristic (ROC) curves were employed to compare the predictive value of PKM2 and a cluster of conventional risk factors by measuring the area under the curve (AUC). The net reclassification improvement (NRI) was utilized to evaluate the enhancement in risk prediction upon the inclusion of PKM2 in a predictive model containing a cluster of conventional risk factors. The integrated discrimination improvement (IDI) was examined to assess the improvement in risk prediction by comparing the average predicted probabilities between the models with and without PKM2. All statistical analyses were performed with SPSS 19 (SPSS Inc., Chicago, USA), MedCalc 20.116 (MedCalc Software Ltd, Ostend, Belgium) and R software 3.4.0 (R foundation, Indianapolis, USA) with the packages “survIDINRI” and “survC1”. All probabilities were 2-sided and P < 0.05 were reported as statistically significant.

## Results

### Baseline characteristics

The demographic, clinical and laboratory characteristics of controls, CCS patients and ACS patients were shown in Table 1. There were no significant differences observed among the three groups in terms of age, sex, BMI, Crea concentrations, blood pressure, and left ventricular end diastolic dimension (LVED). However, significant differences were found among three groups in WBC, FBG, TC, TG, LDL-c, HDL-c, BUN, UA, NT-proBNP, Hs-CRP, Hcy, left ventricular ejection fractions (LVEF) and the presence of smoking, diabetes and hypertension.

**Table 1:** Baseline clinical characteristics and PKM2 concentrations

**Variables**	**Controls** **(N = 238)**	**CCS patients** **(N = 690)**	**ACS patients** **(N = 1753)**	**P**
Age (years)	64 (22-88)	65 (23-87)	65 (27-94)	0.430
Male, N (%)	174 (73%)	538 (78%)	1359 (78%)	0.272
BMI (kg/m^2^)	24 (22-26)	25 (22-27)	25 (22-27)	0.136
SBP (mmHg)	130 (121-144)	132 (120-145)	129 (114-143)	0.053
DBP (mmHg)	78 (70-85)	80 (72-87)	79 (70-88)	0.307
WBC (x10^9^/L)	7.5 (5.0-10.5)	6.8 (5.5-8.3)	8.7 (6.7-11.5)	< 0.001
FBG (mmol/L)	5.7 (5.1-6.1)	5.9 (5.2-7.2)	6.1 (5.3-7.3)	< 0.001
TC (mmol/L)	4.5 (3.7-5.4)	4.5 (3.8-5.2)	4.7 (4.0-5.5)	0.001
TG (mmol/L)	1.6 (1.1-2.1)	1.8 (1.4-2.4)	1.8 (1.4-2.4)	< 0.001
LDL-c (mmol/L)	2.8 (2.0-3.7)	2.9 (2.3-3.7)	3.1 (2.5-3.9)	< 0.001
HDL-c (mmol/L)	1.0 (0.9-1.2)	1.0 (0.8-1.1)	0.9 (0.8-1.1)	< 0.001
BUN (mmol/L)	4.7 (4.0-5.8)	5.2 (4.2-6.5)	5.2 (4.2-6.6)	< 0.001
Crea (μmol/L)	75 (66-89)	77 (66-93)	77 (66-90)	0.279
UA (μmol/L)	336 (283-400)	362 (302-429)	356 (294-431)	0.002
NT-proBNP (ng/L)	125 (53-271)	225 (65-880)	401 (98-1293)	< 0.001
hs-CRP (mg/L)	1.1 (0.7-1.9)	3.3 (1.0- 9.1)	6.4 (1.8-15.8)	< 0.001
Hcy (μmol/L)	9.0 (7.4-11.2)	9.4 (7.8-12.1)	9.7 (7.6-12.5)	0.012
LVEF (%)	61.10 (56.40-66.30)	61.20 (55.60-67.20)	56.20 (46.00-63.20)	< 0.001
LVED (mm)	47.60 (44.70-51.20)	48.50 (45.00-52.00)	48.10 (45.00-52.00)	0.074
**Cardiovascular risk factors**				
Smoking, N (%)	102 (43%)	372 (55%)	1017 (58%)	< 0.001
Diabetes, N (%)	5 (2.1%)	268 (39%)	644 (37%)	< 0.001
Hypertension, N (%)	85 (36%)	457 (66%)	1096 (63%)	< 0.001
**Cardiovascular medication**				
Statins, N (%)	74 (31%)	595 (86%)	1499 (86%)	< 0.001
ACEI/ARB N (%)	81 (34%)	398 (58%)	946 (54%)	< 0.001
β-blocker N (%)	85 (36%)	381 (55%)	881 (50%)	< 0.001
PKM2 concentrations (ng/mL)	0.27 (0.18-0.36)	0.45 (0.23-0.71)	0.52 (0.32-0.81)	< 0.001
Age is reported as median (min-max). Other values are presented as median value (interquartile range) or N (%).PKM2 - pyruvate kinase M2. ACS - acute coronary syndrome. CCS - chronic coronary disease. BMI - body mass index. SBP - systolic blood pressure. DBP - diastolic blood pressure. WBC - white blood cell. FBG - fasting glucose. TC - total cholesterol. TG - triglycerides. LDL-c - low density lipoprotein cholesterol. HDL-c - high density lipoprotein cholesterol. BUN - blood urea nitrogen. Crea – creatinine. UA - uric acid. NT-proBNP - N-terminal pro-brain natriuretic peptide. hs-CRP - high sensitive C reactive protein. Hcy - homocysteine. LVEF - left ventricular ejection fractions. LVED - left ventricular end diastolic dimension. ACEI - angiotensin converting enzyme inhibitor. ARB - angiotensin receptor blocker. P < 0.05 was considered statistically significant.				

### Predictors of ACS among CAD patients

Coronary artery disease patients had significantly higher plasma PKM2 concentrations compared with controls (0.51 (0.30-0.78) ng/mL *vs*. 0.27 (0.18-0.36) ng/mL, P < 0.001). We aimed to identify predictors that could be more relevant in the acute presentation of CAD. Among CAD patients, those with ACS showed higher plasma PKM2 concentrations compared to patients with CCS (0.52 (0.32-0.81) ng/mL *vs*. 0.45 (0.23-0.71) ng/mL, P < 0.001; Table 1). Univariate logistic regression analysis revealed an association between the presence of ACS and several factors including WBC, TC, TG, LDL-c, Hs-CRP, LVEF, and PKM2 concentrations (Table 2). All univariable factors with P < 0.05 entered the stepwise backward multivariable regression analysis. Multivariate logistic regression revealed that baseline plasma PKM2 concentrations are predictors of ACS in CAD patients (odds ratio (OR) 2.17, 95% confidence intervals (CI): 1.63 to 2.89, P < 0.001; Table 2).

**Table 2:** Logistic regression analysis for the presence of ACS in CAD patients

**Variables**	**Univariate regression** **OR (95% CI)**	**P**	**Multivariate regression** **OR (95% CI)**	**P**
WBC (x10^9^/L)	1.22 (1.18-1.25)	< 0.001	1.19 (1.15-1.22)	< 0.001
TC (mmol/L)	1.20 (1.11-1.30)	< 0.001	1.11 (0.98-1.26)	0.096
TG (mmol/L)	1.09 (1.01-1.18)	0.034	1.05 (0.96-1.15)	0.258
LDL-c (mmol/L)	1.22 (1.12-1.33)	< 0.001	1.02 (0.92-1.13)	0.760
hs-CRP (mg/L)	1.01 (1.00-1.01)	< 0.001	1.00 (0.99-1.00)	0.149
LVEF (%)	0.97 (0.96-0.98)	< 0.001	0.98 (0.97-0.99)	< 0.001
PKM2 (ng/mL)	2.27 (1.72-2.99)	< 0.001	2.17 (1.63-2.89)	< 0.001
ACS - acute coronary syndrome. CAD - coronary artery disease. OR - odds ratio. CI - confidence interval. WBC - white blood cell. TC - total cholesterol. TG – triglycerides. LDL-c - low density lipoprotein cholesterol. hs-CRP - high sensitive C reactive protein. LVEF - left ventricular ejection fractions. PKM2 -pyruvate kinase M2. P < 0.05 was considered statistically significant.				

### Correlation of plasma PKM2 concentrations with SYNTAX scores

Among CAD patients, the Spearman correlation analysis indicated a statistically significant correlation between plasma PKM2 concentrations and SYNTAX scores (r_s_ = 0.17, P < 0.001).

### Survival analyses

**Figure 1:**
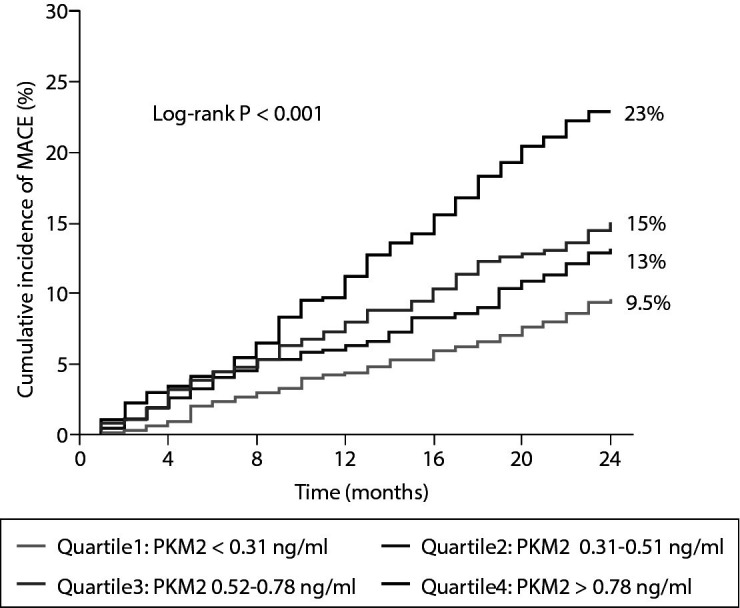
The cumulative incidence of major adverse cardiovascular events (MACEs) is depicted according to pyruvate kinase M2 (PKM2) distribution in quartiles.

Among CAD patients, 368 (15%) MACE were identified during the 2 years follow-up. Cumulative incidence of MACEs based on PKM2 distribution in quartiles is demonstrated in Figure 1. The incidence of MACEs was 9.5% in the first quartile compared to 13%, 15% and 23% in the second, third and fourth quartile, respectively. Kaplan-Meier curves revealed significant differences in the incidence of MACEs among patients with different PKM2 concentrations (Log-rank P < 0.001, Figure 1). Cox proportional hazards models were applied for the development of MACEs according to PKM2 concentrations as quartiles. As shown in Table 3, both crude and adjusted models showed significantly increased risks for development of MACEs in the higher quartile of PKM2 compared with in the lower.

**Table 3:** Hazard ratios and 95% confidence intervals for MACEs according to PKM2 distribution in quartiles in Cox proportional hazards models

	**HR (95% CI) by Quartiles**				
**Variables**	**Quartiles 1**	**Quartiles 2**	**Quartiles 3**	**Quartiles 4**	**P**
Crude	1.0 (reference)	1.39 (0.99-1.95)	1.73 (1.25-2.39)	2.64 (1.95-3.58)	< 0.001
Model 1	1.0 (reference)	1.39 (0.99-1.94)	1.729 (1.25-2.39)	2.637 (1.95-3.57)	< 0.001
Model 2	1.0 (reference)	1.39 (0.99-1.95)	1.75 (1.26-2.42)	2.68 (1.97-3.63)	< 0.001
Model 3	1.0 (reference)	1.39 (0.99-1.94)	1.75 (1.26-2.42)	2.69 (1.98-3.64)	< 0.001
Quartiles 1: PKM2 < 0.31 ng/mL. Quartiles 2: PKM2 0.31 - 0.51 ng/mL. Quartiles 3: PKM2 0.52 - 0.78 ng/mL. Quartiles 4: PKM2 > 0.78 ng/mL. Model 1: adjusted for age and sex. Model 2: adjusted for Model 1 plus traditional risk factors including TC, TG, LDL-c, UA, NT-proBNP, Hs-CRP and Hcy. Model 3: adjusted for Model 2 plus presence of smoking, diabetes and hypertension. P < 0.05 was considered statistically significant. HR - hazard ratio. MACE - major adverse cardiovascular event. PKM2 - pyruvate kinase M2. TC - total cholesterol, TG - triglycerides. LDL-c - low density lipoprotein cholesterol. UA - uric acid. NT-proBNP - N - terminal pro-brain natriuretic peptide. hs-CRP - high sensitive C reactive protein. Hcy - homocysteine.					

### ROC curve analysis

**Figure 2:**
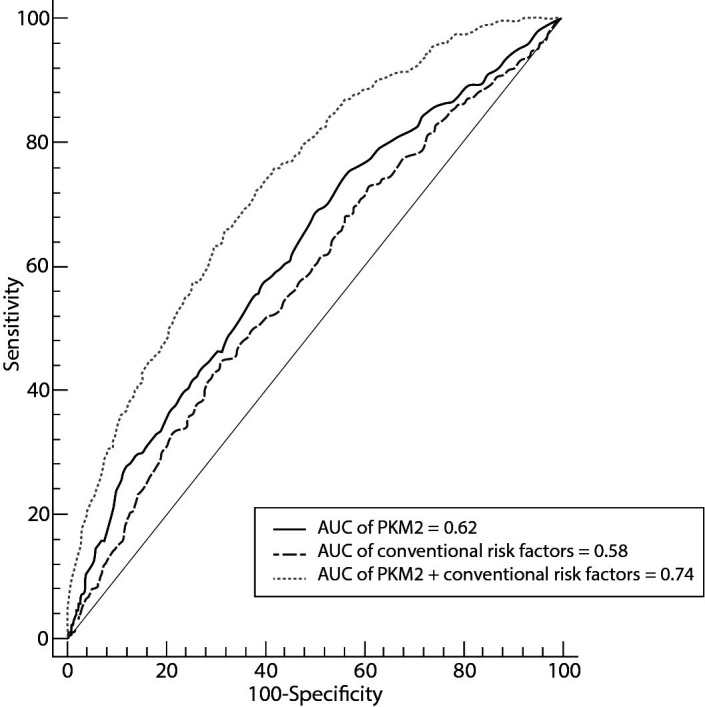
Comparison of area under the curve (AUC) for development of major adverse cardiovascular events (MACEs) between pyruvate kinase M2 (PKM2) and conventional cardiovascular risk factors. Solid line - identity line.

Receiver operating characteristic curves were evaluated to determine whether PKM2 concentration could predict MACEs among CAD patients. Figure 2 shows that the cut-off value of PKM2 for predicting occurrence of MACEs is 0.48 ng/mL, with a reported sensitivity of 0.69 and specificity of 0.50. The positive likelihood ratio (LR+) is 1.38, and the negative likelihood ratio (LR-) is 0.62. We then compared the predictive value of PKM2 with that of a cluster of conventional risk factors composed with TG, TC, LDL-c, UA, NT-proBNP, hs-CRP and Hcy by measuring the AUC. There was no significant difference between the AUC of PKM2 and conventional CAD risk factors (0.62, 95% CI: 0.60 to 0.64 *vs.* 0.58, 95% CI: 0.56 to 0.60, P = 0.059, Figure 2). However, the combination of PKM2 with conventional risk factors resulted in a considerable improvement in AUC compared to conventional risk factors alone (0.74, 95% CI: 0.72 to 0.75 *vs*. 0.59, 95% CI: 0.56 to 0.60, P < 0.001, Figure2).

### Performance evaluation of PKM2 in risk prediction

Further, NRI and IDI were utilized to evaluate the enhancement in risk prediction by incorporating PKM2 into a model consisting of conventional risk factors, such as TG, TC, LDL-c, UA, NT-proBNP, hs-CRP, and Hcy. The prognostic value of conventional risk factors was significantly increased by the addition of PKM2, resulting in a continuous NRI of 0.179 (95% CI: 0.017 to 0.238; P < 0.001) and an IDI of 0.024 (95% CI: 0.012 to 0.038; P < 0.001).

## Discussion

This pilot study demonstrated that 1) baseline plasma PKM2 concentration was an independent predictor of ACS in CAD patients; 2) CAD patients with higher plasma PKM2 concentration had higher cumulative incidence of MACEs compared to patients with lower plasma PKM2 concentration; 3) the addition of PKM2 to a cluster of conventional risk factors significantly improved its performance for predicting the development of MACEs. Taken together, these data provide preliminary evidence that plasma PKM2 concentration predicts the clinical severity and prognosis of CAD.

Effective risk stratification empowers healthcare professionals to identify individuals at increased risk of developing CAD or experiencing MACEs. This enables early identification and intervention in high-risk individuals, facilitating timely implementation of preventive measures and treatments. Biomarkers, as a non-invasive alternative, offer a powerful approach for risk stratification and therapeutic monitoring. Reliable biomarkers not only aid in risk assessment but also contribute to a deeper understanding of the underlying pathogenesis of CAD, enabling clinicians to explore potential new therapeutic targets.

We found that plasma PKM2 concentrations were elevated in CAD patients compared with age and gender matched controls. This finding is in accordance with previous studies that demonstrated PKM2 overexpression in generated macrophages and atherosclerotic plaques from patients with CAD (*12*, *19*). As a glycolytic rate-limiting enzyme, PKM2 plays a crucial role in controlling the increased glucose uptake and high energy consumption required for macrophage activation (*3*, *12*). Activation of macrophages accompanied by increased glucose uptake stimulates PKM2 overexpression in the pathophysiological process of atherosclerosis (*12*, *19*). We showed that ACS patients had higher plasma PKM2 concentrations compared with CCS patients. This could be attributed to an acute episode of overwhelming activation and expansion of macrophages in ACS patients. We also demonstrated that the elevated plasma PKM2 concentration was the independent predictor of ACS in CAD patients. Acute coronary syndrome is a more severe clinical subtype of CAD compared to CCS. Our study highlighted the potential significance of elevated PKM2 concentration at baseline as a potential marker for identifying individuals with higher clinical severity and an increased risk of developing ACS among CAD patients. This suggests that measuring PKM2 concentrations could be a valuable tool in risk stratification and identifying patients who may require closer monitoring and more aggressive management strategies to prevent the occurrence of ACS.

Another key finding of this study was that plasma PKM2 concentration predicts future adverse cardiovascular events in CAD patients. Our findings align with a previous study that demonstrated PKM2 expression was upregulated in macrophages during atherosclerosis progression (*19*). It was also observed that deleting PKM2 significantly reduced atherosclerosis in mice, regardless of changes in plasma lipid concentrations (*19*). Pyruvate kinase M2 promotes cholesterol uptake and foam cell formation by regulating cluster of differentiation 36 (CD36) expression and binding and uptake of Ox-LDL (*11*). Furthermore, PKM2 regulates macrophage polarization and inflammatory responses by increasing mRNA expression of several proinflammatory genes, including monocyte chemoattractant protein-1 (MCP-1), interleukin-1β (IL-1β) and tumor necrosis factor-α (TNF-α), and reducing expression of the anti-inflammatory genes arginase 1 (Arg-1) and interleukin-10 (IL-10) (*19*, *20*). These results indicated that the increased plasma PKM2 concentrations in CAD patients may reflect the ongoing inflammation and foam cell accumulation in the atheromatous plaque, which may lead to plaque vulnerability and the occurrence of MACEs.

We assessed how PKM2, combined with conventional risk factors, predicts MACEs in CAD patients. Combining PKM2 with conventional risk factors greatly improved the accuracy of risk prediction models, as shown by significant improvements in AUC, NRI, and IDI. These results highlight the importance of incorporating PKM2 and conventional risk factors for better MACE prediction in CAD patients. It confirms our hypothesis and emphasizes the value of considering PKM2 as a biomarker in clinical practice, beyond conventional risk factors alone.

Our study should be interpreted in the context of several potential limitations. First, the sample size of our study was relatively small. Furthermore, we cannot rule out all residual confounding present as every observational cohort study. Large scale prospective randomized controlled trials are needed to determine the role of PKM2 in the prognosis of CAD patients. Second, we only measured PKM2 concentration at a single point in time, which may not reflect concentrations at future time periods. Third, our findings are tempered by the fact that all the data we collected from single medical center. Lastly, an important limitation of the study is the potential lack of standardization in the measurement method for PKM2. As PKM2 is not widely available as a routine biomarker, the measurement techniques used in the study may vary across different laboratories or research settings. This lack of standardization can introduce variability and potentially affect the reliability and comparability of PKM2 measurements between studies.

In conclusion, baseline plasma PKM2 concentrations predict the clinical severity and prognosis of CAD.

## Data Availability

The data that supports the findings of this study are available on request from the corresponding author upon reasonable request.
